# Pediatric kidney transplant recipients are at an increased risk for dysbiosis

**DOI:** 10.3389/fmicb.2025.1499813

**Published:** 2025-01-30

**Authors:** Gizem Yılmaz, Seha Saygılı, Ayşe Ağbaş, Esra Karabağ Yılmaz, Ahmet Variş, Nur Canpolat

**Affiliations:** ^1^Department of Pediatrics, Cerrahpasa Faculty of Medicine, Istanbul University - Cerrahpasa, Istanbul, Türkiye; ^2^Department of Pediatric Nephrology, Cerrahpasa Faculty of Medicine, Istanbul University - Cerrahpasa, Istanbul, Türkiye; ^3^Diagen Biotechnology, Ankara, Türkiye

**Keywords:** children, dysbiosis, gut, intestine, kidney transplantation, microbiota

## Abstract

**Introduction:**

This study aimed to compare the gut microbiota composition in pediatric kidney transplant (KTx) recipient with that of healthy children.

**Methods:**

This cross-sectional observational study included 30 pediatric KTx recipients aged between 7 and 21 years and 25 healthy children. The gut microbiota was assessed using 16S rRNA gene sequencing, with alpha and beta diversity, as well as all statistical analyses, conducted using the Phyloseq library in the R programming language. Taxonomic profiles were evaluated with QIIME2, and differences in gut microbiota profiles were compared using linear discriminant analysis effect size (LEFSe) with an LDA threshold of >2 and *p* < 0.05.

**Results:**

No significant differences were found in alpha and beta diversity between the KTx recipients and healthy controls. However, KTx recipients exhibited significant alterations in microbiota composition, including higher relative abundances of Verrucomicrobiota at the phylum level, and Akkermansia and Neisseria at the genus level (*p* < 0.05 for all). Conversely, there was a decrease in bacterial genera belonging to the phylum Firmicutes. In addition, KTx recipients with a history of frequent urinary tract infections, diarrhea and reduced GFR showed significant increases in bacterial abundance (*p* < 0.05 for all).

**Discussion:**

Pediatric KTx recipients demonstrated significant alterarions in gut microbiota composition, indicating dysbiosis. Further studies are needed to elucidate the cause-and-effect relationships of these changes and their impact on clinical consequencies and long-term prognosis.

## Introduction

The gut microbiota, much like a fingerprint, is unique to each individual, with variations across the population. It begins to establish at birth and is significantly influenced by dietary habits (Swarte et al., 2020; Flint, 2012). Other factors, such as socioeconomic status, living environment, number of cohabitants, and genetic characteristics also play a substantial role in shaping this personalized structure (Gevers et al., 2012). Additionally, chronic disease and medications (type, frequency, duration, etc.) further contribute to variations in the microbiota (Clemente et al., 2018; Brown et al., 2013; Chang and Lin, 2016; Thaiss et al., 2016).

There is a bidirectional interaction between the gut and the kidneys. Conditions like uremia, oxidative stress, dietary restrictions, and the use of antibiotics all negatively affect the gut flora and cause dysbiosis. As a result of dysbiosis, increased toxins of intestinal origin, escalation of the existing inflammatory process, and disruption of the bacterial balance lead to kidney damage (Yang and Tarng, 2018; Yao et al., 2021; Hoang et al., 2021). Alterations in the gut microbiota has also been linked to a reduced quality of life, increased incidence of various infections, inflammation, and fluctuations in the doses of immunosuppressive agents (Swarte et al., 2020; Lee et al., 2014, 2019).

Previous research indicates changes in the gut microbiota and increased gut permeability in chronic kidney disease (CKD) (Vanholder and Glorieux, 2015; Holle et al., 2022). Kidney transplantation has also been found to induce significant changes in the gut microbiota. Microbiota analyses mostly examine changes in diversity and taxonomic level. Microbial detailed analysis includes alpha diversity, which evaluates the richness and distribution within individual gut samples, and beta diversity, which examines differences in diversity between samples, both highlighting distinct aspects of dysbiosis and bacterial abundance across taxonomic levels, from phylum to species (broad to specific). Recent studies revealed that long-term use of immunosuppressive agents after transplantation and use of various antibiotics, whether for prophylactic or therapeutic purposes, can induce changes in the gut microbiota (Lee et al., 2014; Chong and Koh, 2020). These changes are linked to various problems, either directly or indirectly, such as chronic diarrhea, urinary tract infection (UTI)s, rejections, and alterations in drug levels (Lee et al., 2019; Vanholder and Glorieux, 2015; Holle et al., 2022; Chong and Koh, 2020; Fricke et al., 2014; Winichakoon et al., 2022).

While most studies on dysbiosis in kidney transplant (KTx) recipients have been conducted in adults, pediatric research on this topic has been limited to small numbers of patients, mainly due to cost concerns (Lee et al., 2014). Therefore, this study aims to identify specific changes in the gut microbiota of pediatric KTx recipients and to show potential differences from healthy subjects.

## Materials and methods

### Study population

**Figure 1:**
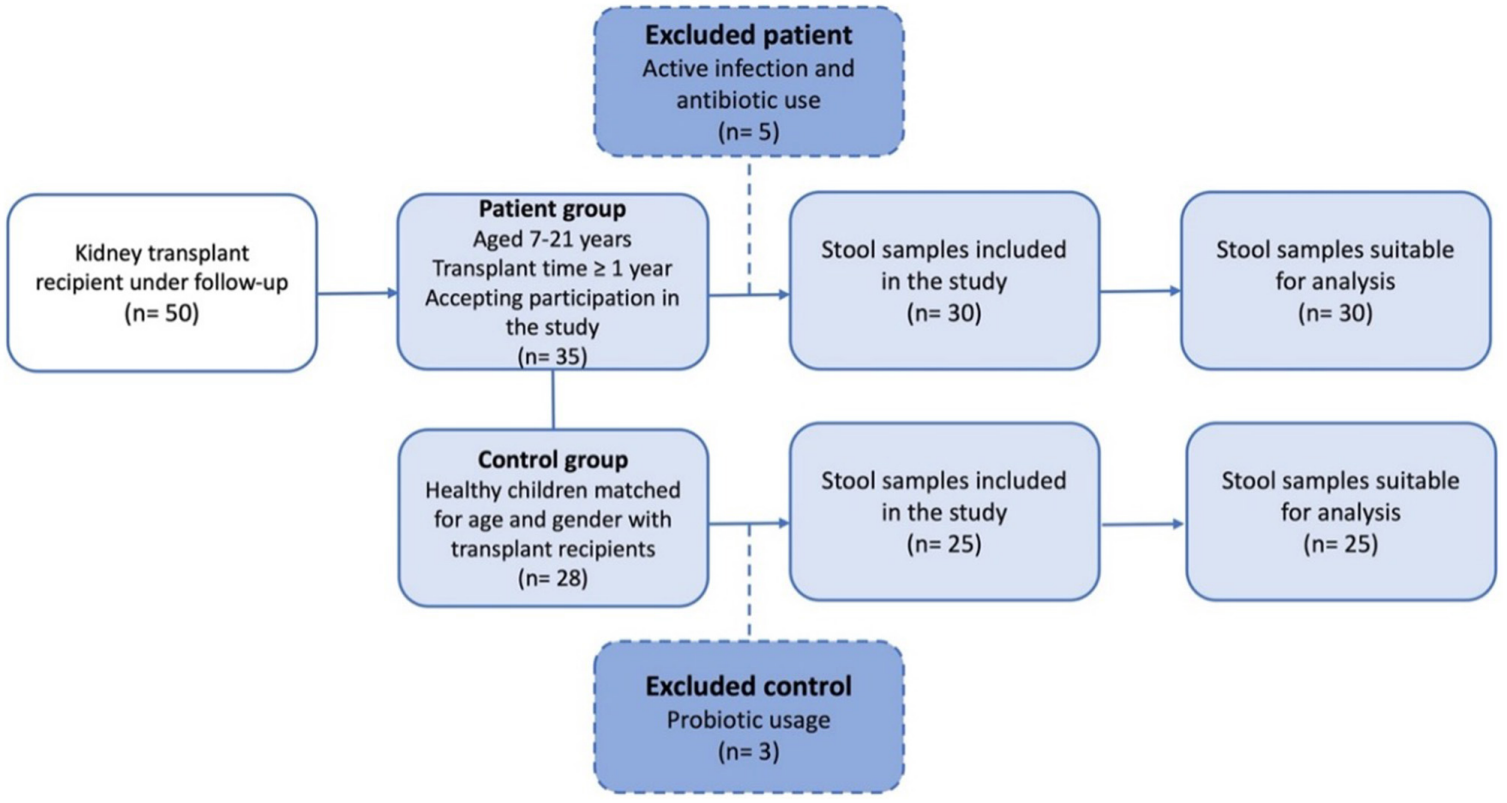
Flowchart of the patient and control group included in the study.

This cross-sectional, observational, single-center study enrolled all KTx recipients aged 7–21 years who were followed up for at least 1 year with a functioning allograft at the Department of Pediatric Nephrology, Istanbul University-Cerrahpasa, Cerrahpasa Faculty of Medicine. Exclusion criteria comprised patients with chronic diseases other than end-stage kidney disease, a history of malignancy and prior chemotherapy, and those who refused to participate in the study. Participants with ongoing gastrointestinal infections or who had received antibiotics for treatment or prophylaxis or probiotic within the previous 6 months were also excluded. Ultimately, the patient group consisted of 30 eligible KTx recipients (Figure 1). The control group consisted of 25 age- and sex- comparable healthy subjects with no known gastrointestinal infections and no use of antibiotics or prebiotics within the previous 6 months.

This clinical study was designed and conducted in accordance with the principles outlined in the Declaration of Helsinki, Good Clinical Practice guidelines, patient rights regulations, and ethical committee approvals. Prior to participation, written informed consent was obtained from both parents and children. Financial support for this study was provided by the Scientific Research Projects Coordination Unit of Istanbul University-Cerrahpasa (TTU-2022-36777).

### Clinical characteristics

Clinical characteristics of the patients including demographics, primary kidney disease, kidney replacement therapy (KRT) features and transplant-related features [transplant age, body mass index (BMI) at the time of transplant, HLA (Human Leukocyte Antigen) tissue compatibility, donor type, induction and maintenance immunosuppressive therapies, history of rejection and its treatment, other medications being used, history of UTI and prolonged diarrhea during posttransplant period] were recorded from patient files.

Anthropometrics measurements (weight and height) were taken at the time of the study for both KTx recipients and healthy controls and then BMI was calculated based on these measurements. The standard deviation scores (SDS) of all anthropometric parameters were calculated using the national references (Neyzi et al., 2015). Obesity was defined as a BMI (according to height-for-age) equal to or above the 95th percentile. Urinary tract infection was defined by presence of symptoms, pyuria, along with the presence of the responsible bacteria in the urine culture. Prolonged diarrhea was defined as acute-onset diarrhea persisting for 7 days or longer. Estimated glomerular filtration rate (eGFR)—the volume of blood filtered per minute by the renal glomeruli—calculated based on the modified Schwartz formula (Schwartz et al., 2009).

### Stool sample collection

During the study, ~25 mg of stool samples were collected from both patients and healthy controls using sterile spoons and placed into sterile containers. The central portion of these samples was separated into two different sterile containers and stored at −80°C without undergoing any processing. All collected samples were then delivered to the laboratory where microbiota analysis.

### DNA extraction

DNA extraction from stool samples was performed using the DiaRex^®^ Stool Genomic DNA Extraction Kit. 250 μl stool lysis solution (SLD) was added to 25–50 mg stool samples. Homogenization of the samples was carried out using ~10 zirconium beads in Fastprep FP120 instrument at 4,000 rpm 2 × 20 s protocol. After homogenization, 25 μl Proteinase K (PKD) was added to mix and incubated at 56°C for 60 min. The solution was then centrifugated at 5000g for 5 min and the supernatant was transferred to a new microcentrifuge tube. 200 μl Lysis Buffer (LBD) was added to the supernatant and the solution was incubated at 70°C for 10 min. After incubation process, 250 μl of absolute ethanol was added to the solution and the whole volume was transferred to columns. The column was centrifuged at 8,000 g for 1 min and then washed according to the manufacturer's protocol. After the last wash, 100 μl Elusion (EBD) was added to the column and incubated at room temperature for 2 min. The eluted DNA was then collected in a new sterile microcentrifuge tube by centrifugation at 8,000 g for 1 min.

### 16S amplicon sequencing

Before 16S rRNA sequencing was performed, stool samples were delivered to authorized laboratory personnel using cooling systems to ensure sample integrity. 16S V3-V4 regions were amplified using the protocol published by Illumina (16S Metagenomic Sequencing Library Preparation, illumina, Part # 15044223 Rev. B) (Klindworth et al., 2013). Amplified and indexed libraries were purified using AmpureXP beads (Beckman Coulter, USA) according to the manufacturer's instructions. After purification, the amount of library was measured using Qubit 3.0 (Therm Fisher, USA) and the molarity of the library was calculated using the algorithm provided by illumina following the same protocol as above. Following the protocol, the samples were normalized to 4 nM and pooled. The pooled library was added to the illumina reagent kit according to the manufacturer's instructions.

### Statistical analysis

In this study, descriptive data for categorical variables were presented as numbers and percentages (n, %), while continuous variables were expressed as mean (± SDS) or median (25th percentile-75th percentile). Before proceeding with the analysis of continuous variables, the Shapiro-Wilk test was applied to determine whether they followed a normal distribution. In cases where the data did not exhibit normal distribution, the Mann-Whitney U test was employed for binary group comparisons with linear variables, and the Kruskal-Wallis test was used for three or more group comparisons. The chi-square test was used to compare categorical variables.

To assess how continuous variables co-varied, correlation analysis was performed. In cases where the data did not demonstrate a normal distribution, Spearman correlation analysis was preferred. Throughout all statistical analyses, a significance level of *p* < 0.05 was accepted within the framework of 5% Type I error and a 95% confidence interval. The study data were evaluated using the Statistical Package for Social Sciences (SPSS) for Windows version 20.0 (SPSS Inc., Chicago, IL, USA) software program.

### Bioinformatics analysis

Pair-end Illumina reads (2 × 250) were imported into the qiime2 environment (Bolyen et al., 2019). None of the samples were removed from the study. Quality clipping, chimera detection, and cleaning of reads were implemented through the Qiime2 Dada2 pipeline (via q2-dada2) (Callahan et al., 2016). Bases with low phred scores (<Q30) were cut out. Amplicon Sequence Variants (ASV) generated by Dada2 were mapped to the Silva 138 database (https://www.arb-silva.de/documentation/release-138/) (Schloss, 2021; Werner et al., 2012). The Phyloseq object was generated from qiime2 artifact files in the R 4.1 environment (McMurdie and Holmes, 2013). The outcome measures obtained include gut microbiome composition, such as α-diversity and β-diversity, and bacterial abundance at the taxonomic levels of phylum, family, genus and species (from broader to more specific levels, respectively). Alpha diversity assessment, which was used to evaluate the individual expression of richness and distribution in the gut environment of the samples, was interpreted using three different indices, including Chao1, Shannon, and Simpson. *P* values between groups were calculated using Kruskal-Wallis test (Kruskal and Allen, 1952). Beta diversity analysis, which show the diversity values relative to each other among fecal samples, was calculated based on Jaccard, Bray-Curtis, weighted and unweighted Unifrac (Lozupone and Knight, 2005). Specific differences between groups were determined by differential abundance analysis, Deseq2 R package (Love et al., 2014). Linear discriminant analysis Effect Size (LEfSe) identifies biomarkers by comparing groups, using Linear Discriminant Analysis (LDA) to rank features by their contribution to group differences and was conducted between groups to show statistically significant taxonomies (Lozupone and Knight, 2005; Lozupone et al., 2007; Segata et al., 2011). Krona charts are multilevel pie charts used to visualize both the most abundant organisms and their specific classifications (Ondov et al., 2013).

## Results

### Demographic and clinical characteristics

The median age of the 30 pediatric KTx recipients was 16 (7.4–20.8) years, with an equal distribution of 15 females and 15 males. The median age at the start of KRT was 5.6 years, ranging from 1 month to 15.2 years. As pretransplant KRT, hemodialysis was performed in 12 patients (40%), peritoneal dialysis in 17 patients (57%), and pre-emptive kidney transplantation in 1 patient (3%). The median age at transplantation was 9.4 (2.6–17.4) years. Thirteen patients (43%) received a kidney from a living donor and 17 (57%) from a cadaveric donor.

The median time after transplantation was 6.7 years, ranging from 1.1 to 14.4 years. Eleven patients (36.6%) were in 1–5 years post-transplant, 14 patients (46.6%) were 5–10 years post-transplant, and five patients (16.6%) were more than 10 years post-transplant. As part of their immunosuppression regimen, all patients received a steroid-based triple combination therapy. The most regimen was the combination of was steroid, mycophenolate mofetil, and a calcineurin inhibitor in 80% of the patients (*n* = 24) (Table 1). A total of six patients had a history of rejection; two of these patients had two episodes of rejection and the other four had one episode. All rejections were T cell-mediated and none occurred within 6 months prior to the study. During the post-transplant follow-ups, 14 patients (46%) had at least one episode of prolonged diarrhea, and 16 patients (53%) experienced recurrent UTIs. Twelve patients (40%) exhibited impaired kidney function, as indicated by a glomerular filtration rate (GFR) of <60 mL/min/1.73 m^2^. Among these, only one patient had an estimated GFR of <15 mL/min/1.73 m^2^.

**Table 1:** Demographics and clinical characteristics of the study population^*^.

	**KTx recipients n = 30**	**Healthy controls n = 25**
Age, years	16 (7.4–20.8)	15.6 (7.6–17.7)
Male/female, *n* (%)	15 (50)/15 (50)	10 (40)/15 (60)
BMI, kg/m^2^	20.7 (13.6–39)	24.4 (16.2–29.6)
BMI-SDS	−0.1 (−2.5–4.0)	1.0 (−0.7–1.83)
Age at start of KRT, years	5.6 (0.1–15.2)	
Pre-transplant KRT duration, years	2.05 (0–8.6)	
**Pre-transplant KRT type**, ***n*** **(%)**		
Haemodialysis	12 (40)	
Peritoneal dialysis	17 (57)	
Pre-emptive transplant	1 (3)	
**HLA compatibility**, ***n*** **(%)**		
0–1 missmatch	2 (7)	
2–3 missmatch	28 (93)	
4–6 missmatch	0 (0)	
Donor type, living/cadaveric, *n* (%)	13 (76)/17 (24)	
Age at transplant, years	9.4 (2.6–17.4)	
Post-transplant period, years	6.7 (1.1–14.4)	
**Immunsupressive medication**, ***n*** **(%)**		
Steroid	30 (100)	
MMF	27 (90)	
Calcineurin inhibitors	26 (87)	
Everolimus	6 (20)	
Leflunomide	1 (3)	

* Continuous variables are given as median (range).

BMI, body mass index; BMI-SDS, body mass index standard deviation score; KRT, renal replacement therapy; HLA, human leucocyte antigen.

When compared to healthy controls, the KTx recipients did not show significant differences in age, sex, BMI, or BMI-SDS (*p* > 0.05 for all). There were two obese individuals among the KTx recipients (6.6%), but none in the control group (*p* = 0.29). The demographic and clinical characteristics of the study population are shown in Table 1.

### Gut microbiota profile

Alpha diversity analyses revealed that the amplicon sequence variants (ASV) values were higher in the KTx recipients than in the healthy controls, but the differences were not statistically significant for the Chao-1, Simpson, or Shannon indices (*p* > 0.05) (Supplementary Figure 1). Beta diversity analyses revealed distinct clusters in both weighted and unweighted UniFrac analyses, but no significant differences were found between the KTx and control groups (Supplementary Figure 2). A comparative analysis of bacterial abundance (%) between the two groups is also displayed in a Krona chart (Supplementary Figures 3 and 4).

**Figure 2:**
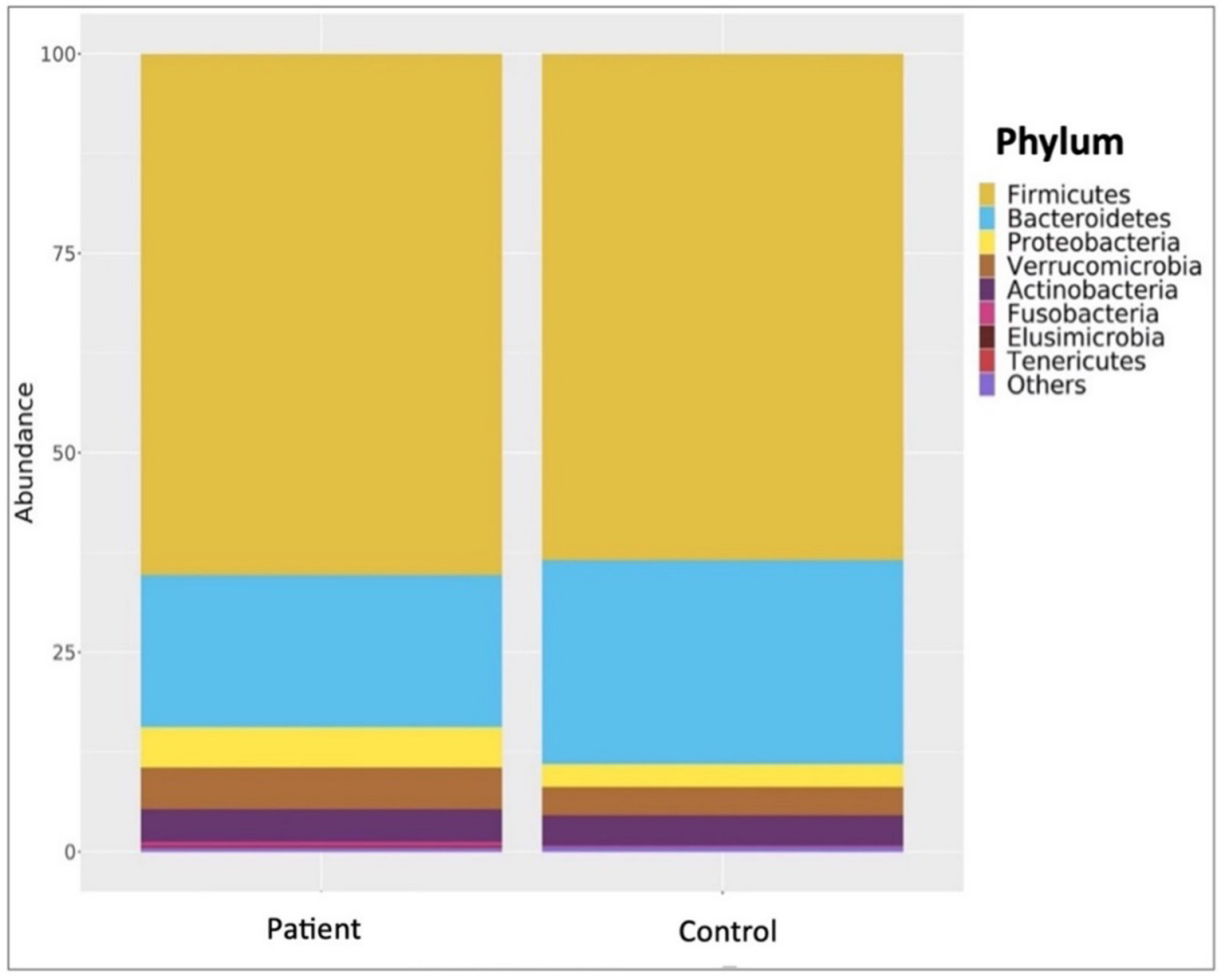
Comparisons of patient and control groups at phylum level. In the KTx group, Firmicutes (62.2%), Bacteroidetes (23.6%), Proteobacteria (4.9%), Actinobacteria (3.4%), and Verrucomicrobia (5.1%) were detected. In the control group, Firmicutes (64.9%), Bacteroidetes (24.1%), Verrucomicrobia (4.5%), Actinobacteria (3.8%) and Proteobacteria (2.2%) were present. The significantly higher abundance of Verrucomicrobiota (p = 0.022) in the transplanted group is shown by taxonomic column plots.

**Figure 3:**
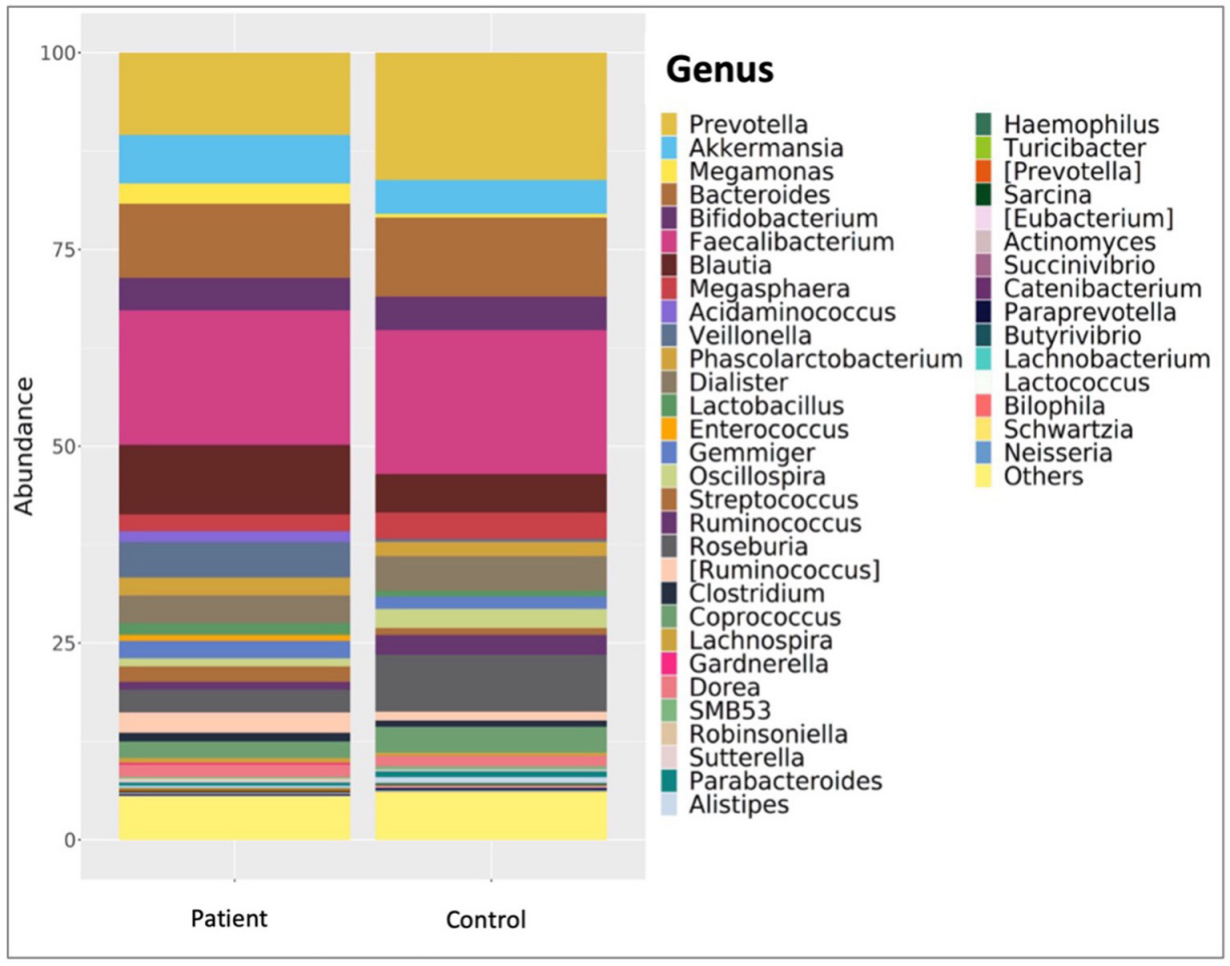
Comparison of patient and control group at genus level. In the KTx group, Faecalibacterium (17.5%), Prevotella (14.3%), Bacteroides (10.6%), Blautia (9%) and Akkermansia (6%) were detected. In the control group, Faecalibacterium (17.3%), Prevotella (15.2%), Akkermansia (5.5%), Bacteroides (9.2%) and Blautia (5%) were present. The high abundance of Akkermansia (p = 0.022) and Neisseria (p = 0.034) genera in the patient group is shown by taxonomic column plots.

**Figure 4:**
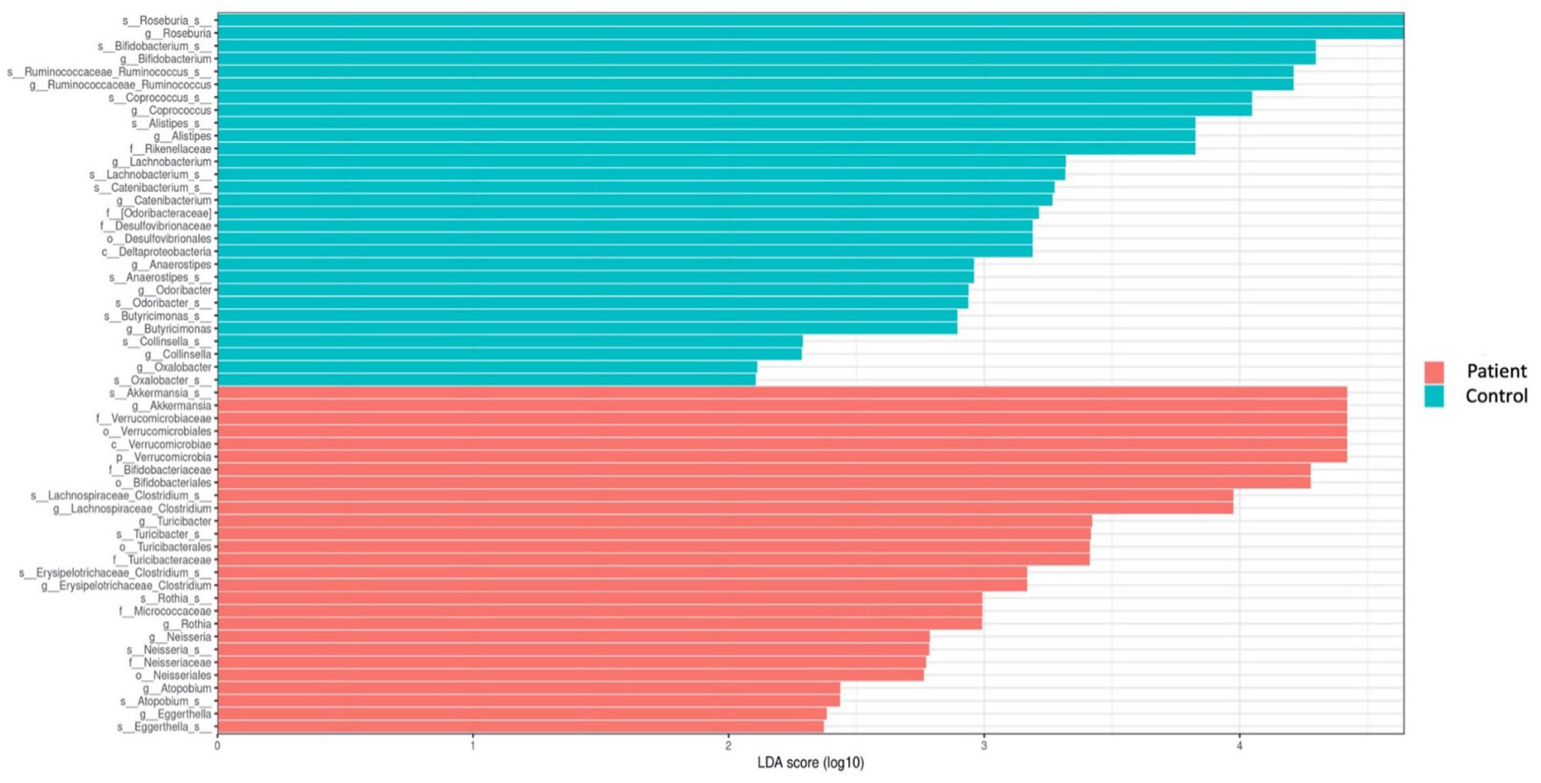
Comparison of taxonomic data of the patient and control groups by the LEFSe analysis (a threshold value of LDA > 2, p < 0.05). Kidney transplant recipients exhibited a significant increase in Verrucomicrobia (*p* = 0.022) at the phylum level and significant increases in Turicibacteraceae (*p* = 0.02), Micrococcaceae (*p* = 0.002), and Neisseriaceae (*p* = 0.034) at the family level. At the species level, Akkermansia (*p* = 0.022), Clostridium (*p* = 0.01), Turicibacter (*p* = 0.02), Rothia (*p* = 0.002), Neisseria (*p* = 0.034), Eggerthella (*p* = 0.04), and Atopobium were found to be dominant among KTx recipients. However, Roseburia (*p* = 0.0007), Ruminococcus (*p* = 0.002), Bifidobacterium (*p* = 0.03), Coprococcus (*p* = 0.02), Alistipes (*p* = 0.001), Catenibacterium (*p* = 0.02), Lachnobacterium (*p* = 0.0009), Anaerostipes (*p* = 0.002), Butyricimonas (*p* = 0.004), and Collinsella (*p* = 0.02) were present at lower levels.

Comparative analyses revealed significant differences between the patient and control groups. At the phylum level, LEfSe analysis revealed a significant increase in Verrucomicrobia (5.1% vs. 4.5%, *p* = 0.022) in KTx recipients, with no significant difference in Firmicutes (62.2% vs. 64.9%, *p* > 0.05), Bacteroidetes (23.6% vs. 24.1%, *p* > 0.05), Proteobacteria (4.9% vs. 2.2%, *p* > 0.05) or Actinobacteria (3.4% vs. 3.8%, *p* > 0.05) (Figure 2). At the genus level, Akkermansia (6% vs. 5.5%, *p* = 0.022) and Neisseria (%0.3 vs. %0 *p* = 0.034) were higher in KTx recipients (Figure 3). However, there were no significant differences in Faecalibacterium (17.5% vs. 17.3%, *p* > 0.05), Prevotella (14.3% vs. 15.2%, *p* > 0.05), Bacteroides (10.6% vs. 9.2%, p >0.05), and Blautia (9% vs. 5%, *p* > 0.05) between the two groups. As shown in Figure 4, the LEfSe analysis, which examines bacterial composition with a threshold value of LDA > 2, also revealed notable changes in the KTx recipients at the phylum, family, and species levels.

**Figure 5:**
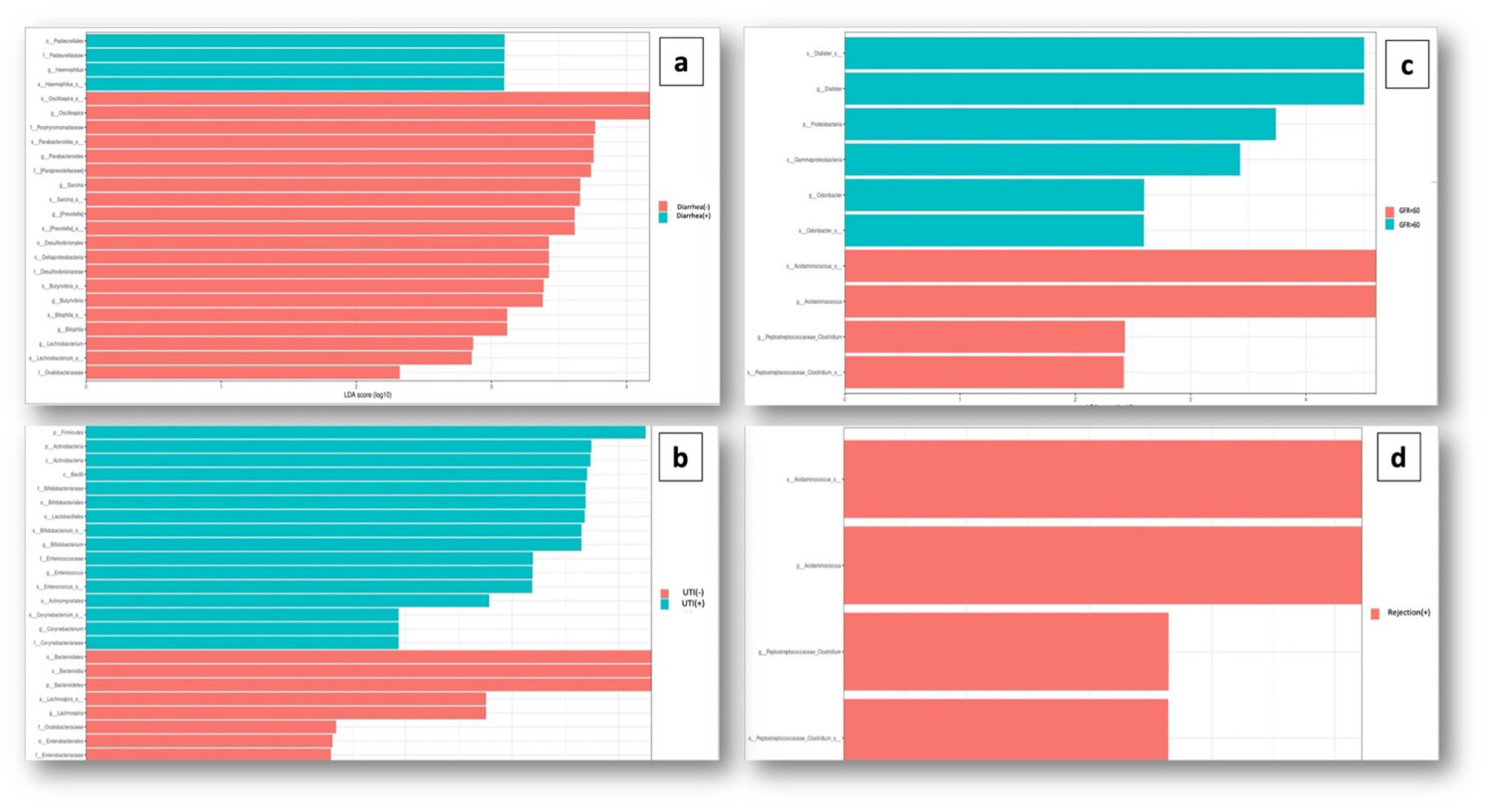
Association of dysbiosis with clinical conditions. **(A)** Significant increase in the Pasteurellaceae family (*p* = 0.01) and the Haemophilus genus (*p* = 0.008) was observed in patients with a history of prolonged diarrhea. **(B)** Significant abundance of bacteria belonging to the Firmicutes phylum (*p* = 0.008), Actinobacteria phylum (*p* = 0.005), Bacilli class (*p* = 0.01), Enterococcus genus (*p* = 0.04), and Corynebacterium genus (*p* = 0.03) was detected In patients with a history of recurrent UTIs. **(C)** Among the patients with impaired kidney function and GFR <60 mL/min/1.73 m^2^, a significant increase in the abundance of Acidaminococcus (*p* = 0.01) and Clostridium genus (*p* = 0.02) bacteria was observed. **(D)** Patients with a history of rejection, an increase in Acidaminococcus (*p* = 0.002) and Clostridium (*p* = 0.03) genera was observed.

When examining the KTx group itself, in 14 patients with a history of prolonged diarrhea, Pasteurellaceae family (*p* = 0.01) and Haemophilus genus (*p* = 0.008) were significantly increased (Figure 5A). Sixteen patients with recurrent UTIs showed higher abundance of Firmicutes phylum (*p* = 0.008), Actinobacteria phylum (*p* = 0.005), Bacilli class (*p* = 0.01), Enterococcus genus (*p* = 0.04), and Corynebacterium genus (*p* = 0.03) (Figure 5B). Among 12 patients with impaired kidney function (estimated GFR < 60 mL/min/1,73m^2^), Acidaminococcus (*p* = 0.01) and Clostridium genus (*p* = 0.02) were significantly increased (Figure 5C). Six patients with a history of rejection had increased Acidaminococcus (*p* = 0.002) and Clostridium (*p* = 0.03) genera (Figure 5D).

## Discussion

The present study revealed significant alterations in the gut microbiota of pediatric KTx recipients, indicating dysbiosis. These alterations included an increase of Verrucomicrobia at the phylum level and in the genera Akkermensia and Neisseria, along with a decrease in certain bacterial genera belonging to the phylum Firmicutes. Additionally, the study also showed that these bacterial changes were pronounced in KTx recipients with a history of recurrent UTIs, diarrhea, and reduced eGFR.

Current literature demonstrated that there are changes in gut microbiota in KTx recipients in both adult and pediatric populations, and there is a relationship between intestinal barrier dysfunction and microbial metabolite imbalance in pediatric CKD and transplant patients (Holle et al., 2022; Tain and Hsu, 2023; Noce et al., 2022; Ferranti et al., 2014). Two different pediatric transplant studies (Table 2) showed a reduction in α-diversity and significant decrease in Bifidobacteria as in our study (Crespo-Salgado et al., 2016). A study involving children with CKD without glomerulonephritis reported an increased abundance of the Verrucomicrobia phylum and Akkermansia genus, alongside a reduced abundance of the Bifidobacterium bifidum species (Swarte et al., 2022). Compatible with these findings, our study showed a significant decrease in Ruminococcus, Coprococcus, Butyricimonas and Bifidobacteria and an increase in the relative abundance of Proteobacteria in post-transplant patients, although not statistically significant. In a study involving adult KTx patients, found increased Proteobacteria and decreased Actinobacteria, especially in bacteria producing butyrate (Swarte et al., 2020). Another study reported increased abundance of Proteobacteria and decreased abundance of Ruminococcus, Coprococcus, and Dorea in the posttransplant population, particularly in cases with diarrhea, and an increased abundance of Enterococcus in KTx patients with a history of UTIs (Lee et al., 2014). A different study involving adult patients at pretransplant and posttransplant weeks 1 and 4, showed Clostridia, Ruminococcus, Faecalibacterium, and Veillonellaceae dominance pre-transplant, and Bacilli and Enterococcus dominance post-transplant (Yu et al., 2021). All these findings reveal that there are changes in gut microbiota in KTx recipients in both adult and pediatric populations.

**Table 2:** Current research involving on pediatric KTx recipients.

**References**	**Number of patients**	**Results**
Holle et al. (2022)	38 children and adolescents with CKD stage 3–5 (12 CKD stage 3–4, 11 HD, 15 KT); 10 controls	Gut barrier dysfunction and microbial metabolite imbalance were associated with a stage-dependent increase in plasma tryptophan metabolites.
Crespo-Salgado et al. (2016)	26 children and adolescents with ESKD (8 HD, 8 PD, 10 KT); 13 controls	α-diversity was decreased in PD and KT, while Bacteroidetes was increased in HD. Plasma indoxyl sulfate and p-cresyl sulfate were elevated in both HD and PD. Firmicutes and Actinobacteria were decreased, and Enterobacteriaceae was increased in PD

Although the significant microbial changes seen in children with kidney transplantation have not been adequately investigated to date, the importance of gut microbiota and the benefits of probiotics have been shown, especially in pediatric inflammatory bowel disease patients (Sun et al., 2024; Huang et al., 2023; Qiu et al., 2022). Similar to the post-transplant gut microbiota changes observed in our study, reported significant decreases in Bifidobacterium longum in ulcerative colitis (UC) and in Eubacterium rectale, Faecalibacterium prausnitzii, and Roseburia intestinalis in both Crohn's disease (CD) and UC, along with an increase in Bacteroides fragilis (Vich Vila et al., 2018). Pittayanon et al. (2020) found an increase in E. coli in UC patients, consistent with our findings. Similarly, showed an increase in Intestinibacter in inflammatory bowel diseases (IBD) and a significant decrease in Coprococcus spp. in CD, also mirroring our results.

A decrease in Faecalibacterium prausnitzii, a sign of inflammation in the gastrointestinal system (Furusawa et al., 2013; Martín et al., 2017; Bruzzese et al., 2014; Sokol et al., 2008; Machiels et al., 2014; Lavelle and Sokol, 2020) aligns with reduced Lachnobacterium and Ruminococceae observed in our patient population, suggesting ongoing inflammatory process at least 1 year after transplantation. Dysbiosis is also attributed to dietary restrictions and long-term use of immunosuppressive and antibiotic medications, with increased Proteobacteria closely linked to dysbiosis (Fricke and Bromberg, 2017; Tourret et al., 2017). A study linked obesity to an increase in the abundance of Firmicutes at the phylum level, Clostridium at the genus level, and Eubacterium rectale, Clostridium coccoides, Lactobacillus reuteri, Akkermansia muciniphila, Clostridium histolyticum, and Staphylococcus aureus at the species level (Gomes et al., 2018). However, the presence of only two obese patients in our study is insufficient to suggest such a relationship.

Bifidobacteria play a crucial role in directing intestinal colonization during the early stages of life, significantly impacting the immune system (Gomes et al., 2018). A decrease in Bifidobacteria, particularly in this critical period, has been linked to compromised immune function in childhood, leading to increased susceptibility to infections (Huda et al., 2019; Wu et al., 2016). Bifidobacteria are also known to provide health benefits through their metabolic activities (Russell et al., 2011). A reduction in Bifidobacteria density has also been observed in patients with diarrhea (Erdogan et al., 2012). The significant decrease observed in our study, especially in the patient group compared to healthy controls, suggests a shift in the intestinal microbiota that may negatively affect the host. This alteration in the gut microbiota was associated with clinical outcomes such as rejection, prolonged diarrhea, frequent UTIs, and the uremic environment in our KTx group. Possible risk factors for dysbiosis were identified, and a more accurate inference can be made by including the intestinal microbiota during infection periods of patients. A study investigating the link between altered microbiomes and conditions like diarrhea, UTI, and graft rejection found that post-transplant patients, especially those with diarrhea, had decreased levels of Ruminococcus, Coprococcus and Dorea species (Lee et al., 2019). The study also showed an increase in Enterococcus abundance in transplant patients with a history of UTI (Lee et al., 2019). Our study similarly showed an increase in Enterococcus in KTx recipients with recurrent UTIs. Additionally, we found an increase in Acidaminococcus and Clostridium in KTx recipients with a history of rejection. Despite the absence of signs of infection in the last 6 months for the KTx recipients included in the study, dysbiosis was detected. These alterations in gut microbiota are attributed to intensive immunosuppressive treatments and prolonged antibiotic use following transplantation (Gabarre et al., 2022). In this context, dysbiosis and the consequent changes in drug levels are considered potential contributors to both rejection and other post-transplant complications. The inclusion of a comparison of the intestinal microbiota between patients in the post-transplant acute and chronic periods in the study could provide more comprehensive assessments.

This single-center and case-control study with the largest cohort to date is one of the few studies focusing on the gut microbiota in pediatric KTx recipients, providing valuable insights into an under-researched area. The present study also reflects a more stable period in the microbiota composition of patients transplanted for at least 1 year compared to the immediate post-transplant period. However, a major limitation of our study is the constrained budget, which restricted the 16S rRNA metagenomic analysis to only a small sample size (55 samples). This also prevented expansion of the sample size to include pre- and post-transplant acute/chronic periods. In addition, the cross-sectional design and lack of longitudinal data limits the ability to infer causality or to observe changes over time, particularly in the pre- and post-transplant periods. Another limitation is the inability to achieve complete homogeneity across all subjects in the patient and control groups due to various factors affecting the gut microbiota, such as mode of delivery, breastfeeding, ethnic and cultural background, and dietary differences. Nevertheless, efforts were made to mitigate these negative aspects by selecting patient and control groups that were similar in terms of age, gender, and BMI characteristics.

In conclusion, pediatric KTx patients exhibit notable changes in gut microbiota indicative of dysbiosis. While our study found no significant differences in alpha and beta diversity between patients and healthy controls, there was an increase in the Verrucomicrobia phylum and the Neisseria genus, alongside a decrease in the Butyricimonas and Bifidobacteria genera. Understanding the cause-and-effect relationship of these microbial shifts is crucial for developing targeted interventions to improve clinical outcomes and long-term prognosis for these patients. Longitudinal studies involving larger chorts, encompassing pre- and post-transplant periods, are needed to achieve this purpose.

## Data Availability

The raw data supporting the conclusion of this article is not publicly available to protect participant confidentiality and privacy. Requests to access the datasets should be directed to the corresponding author.

## Supplementary material

Supplementary Figure 1 Comparative alpha diversity analyses of patient and control Group. **(A)** Shannon analysis (*p* > 0.05). **(B)** Simpson analysis (*p* > 0.05). **(C)** Chao1 analysis (p > 0.05).

Supplementary Figure 2 Patient and control group comparative beta diversity analyses. **(A)** Unweighted Unifrac analysis (p= 0.49). **(B)** Weighted Unifrac analysis (*p* = 0.35).

Supplementary Figure 3 Krona charts showing bacterial relative abundance in patient group.

Supplementary Figure 4 Krona charts showing bacterial relative abundance in control group.
