# Supplementary figures and images for: Pediatric kidney transplant recipients are at an increased risk for dysbiosis
Source: Front Microbiol. 2025 Jan 30;16:1499813. doi: 10.3389/fmicb.2025.1499813 (PMC11823477; doi:10.3389/fmicb.2025.1499813)

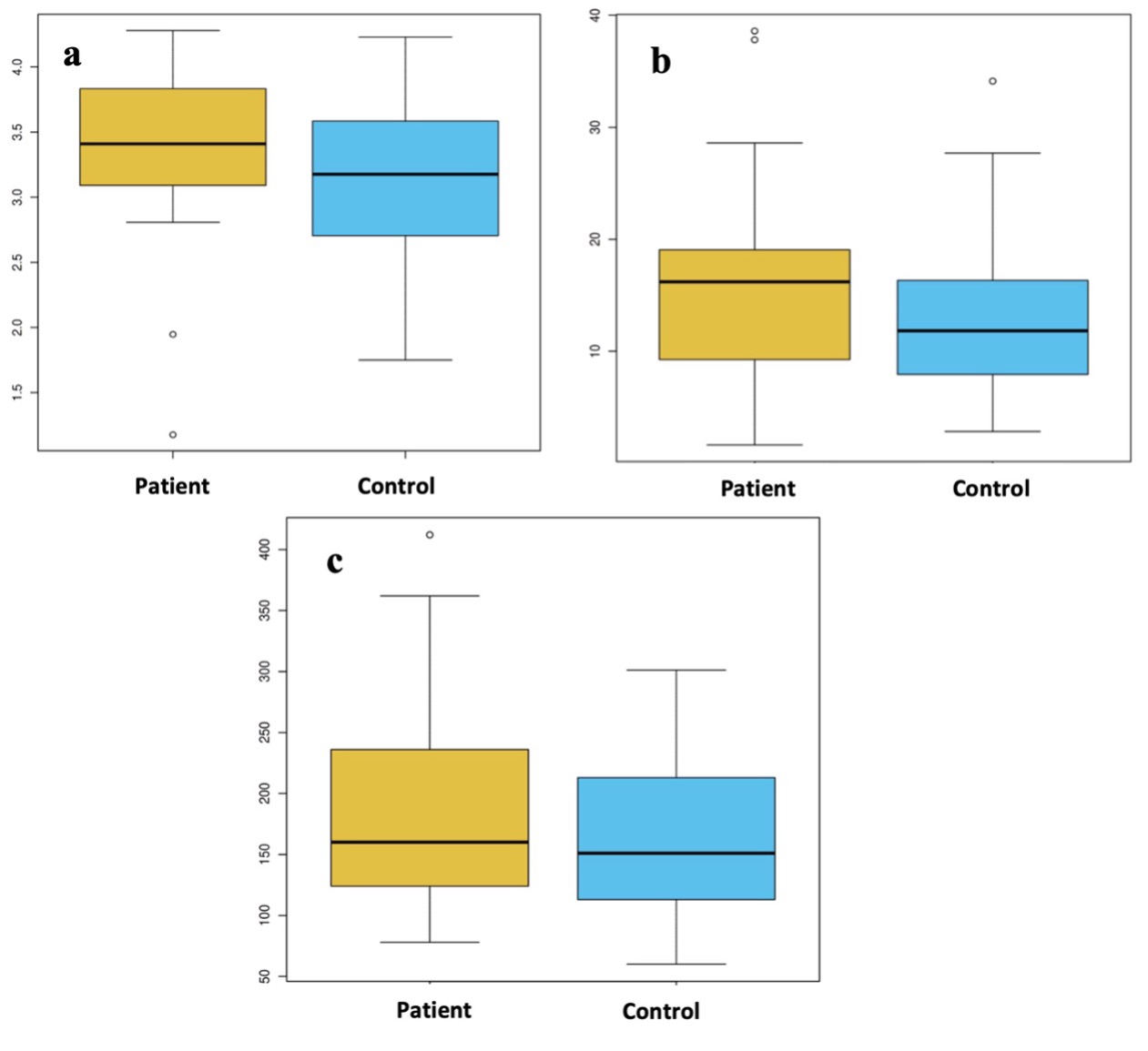

Supplement: Supplementary Figure 1 — Comparative alpha diversity analyses of patient and control Group. (A) Shannon analysis (p > 0.05). (B) Simpson analysis (p > 0.05). (C) Chao1 analysis (p > 0.05). [file Image_1.jpeg]

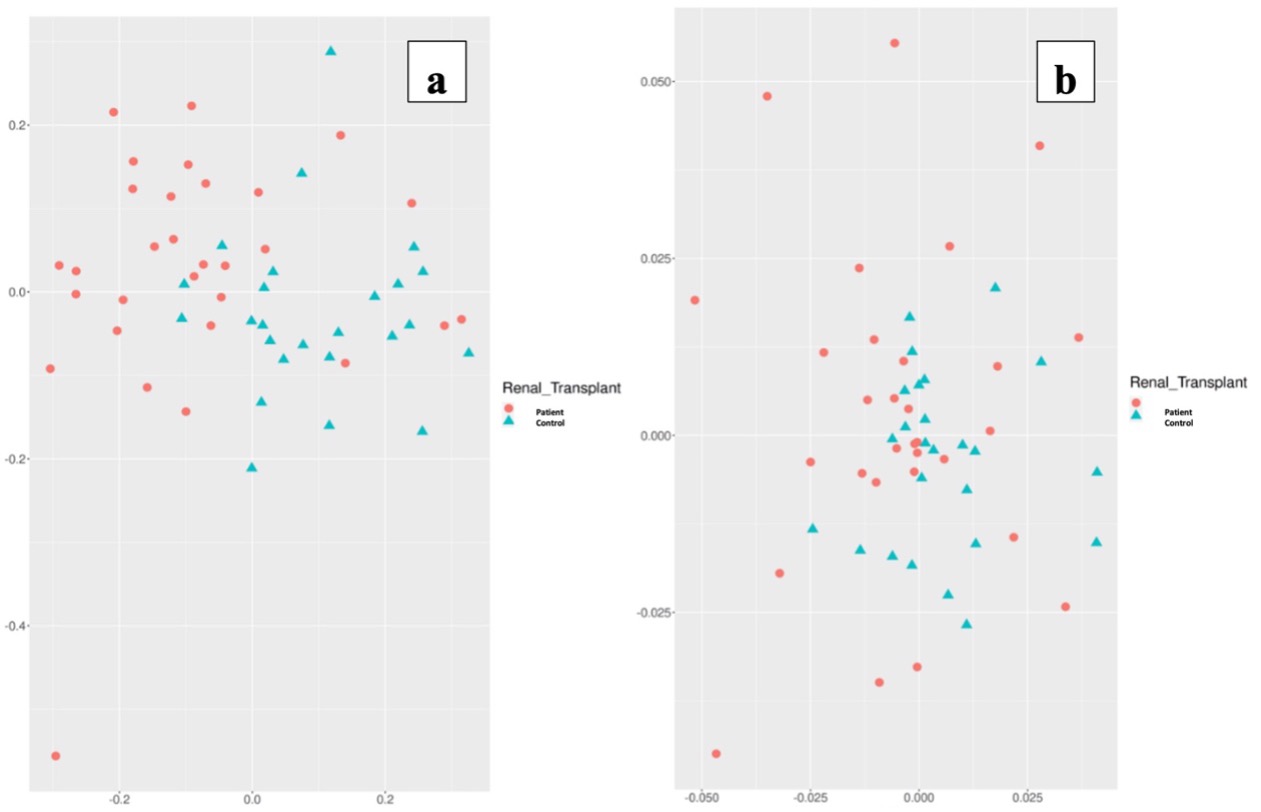

Supplement: Supplementary Figure 2 — Patient and control group comparative beta diversity analyses. (A) Unweighted Unifrac analysis (p= 0.49). (B) Weighted Unifrac analysis (p = 0.35). [file Image_2.jpeg]

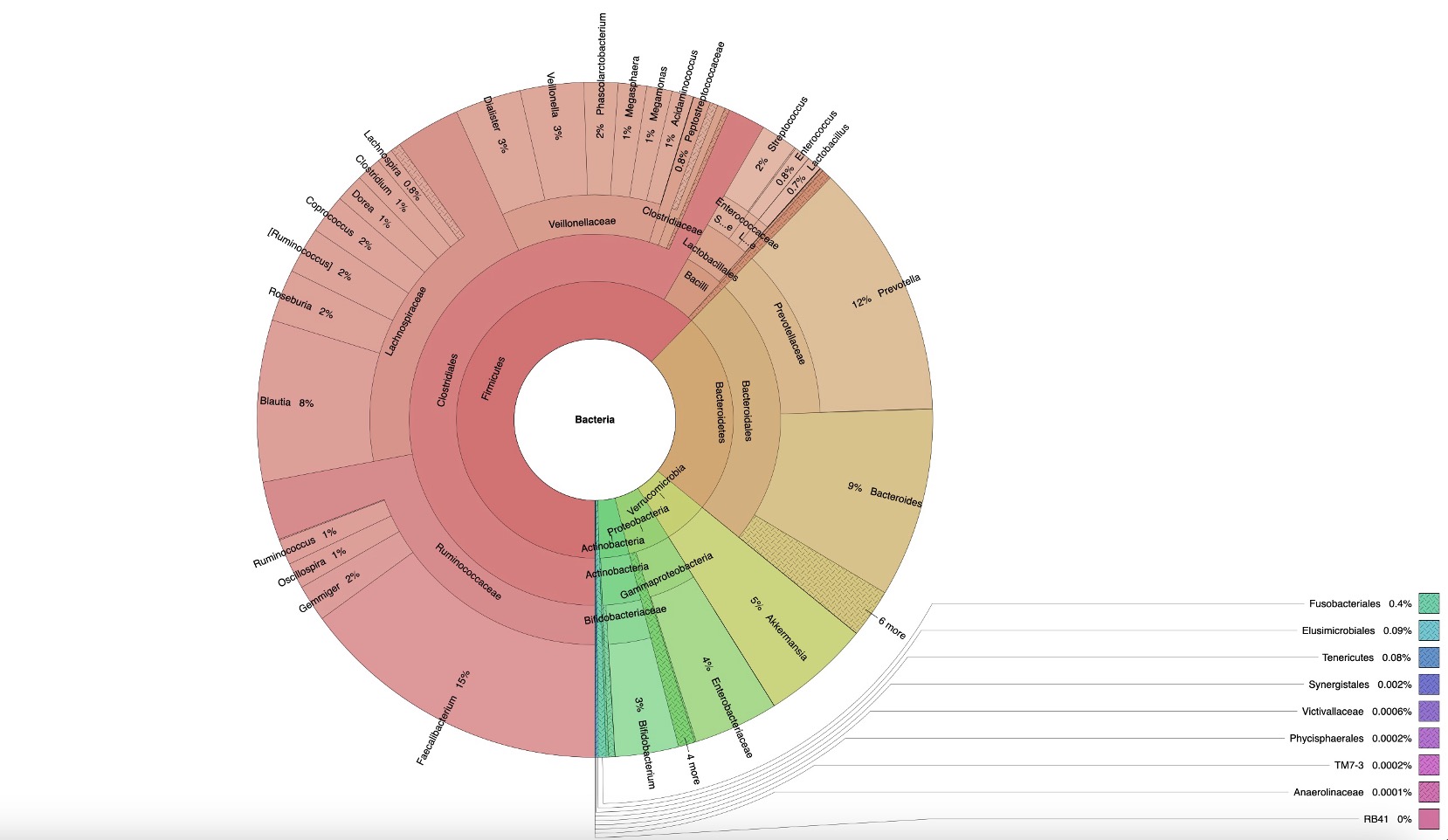

Supplement: Supplementary Figure 3 — Krona charts showing bacterial relative abundance in patient group. [file Image_3.jpeg]

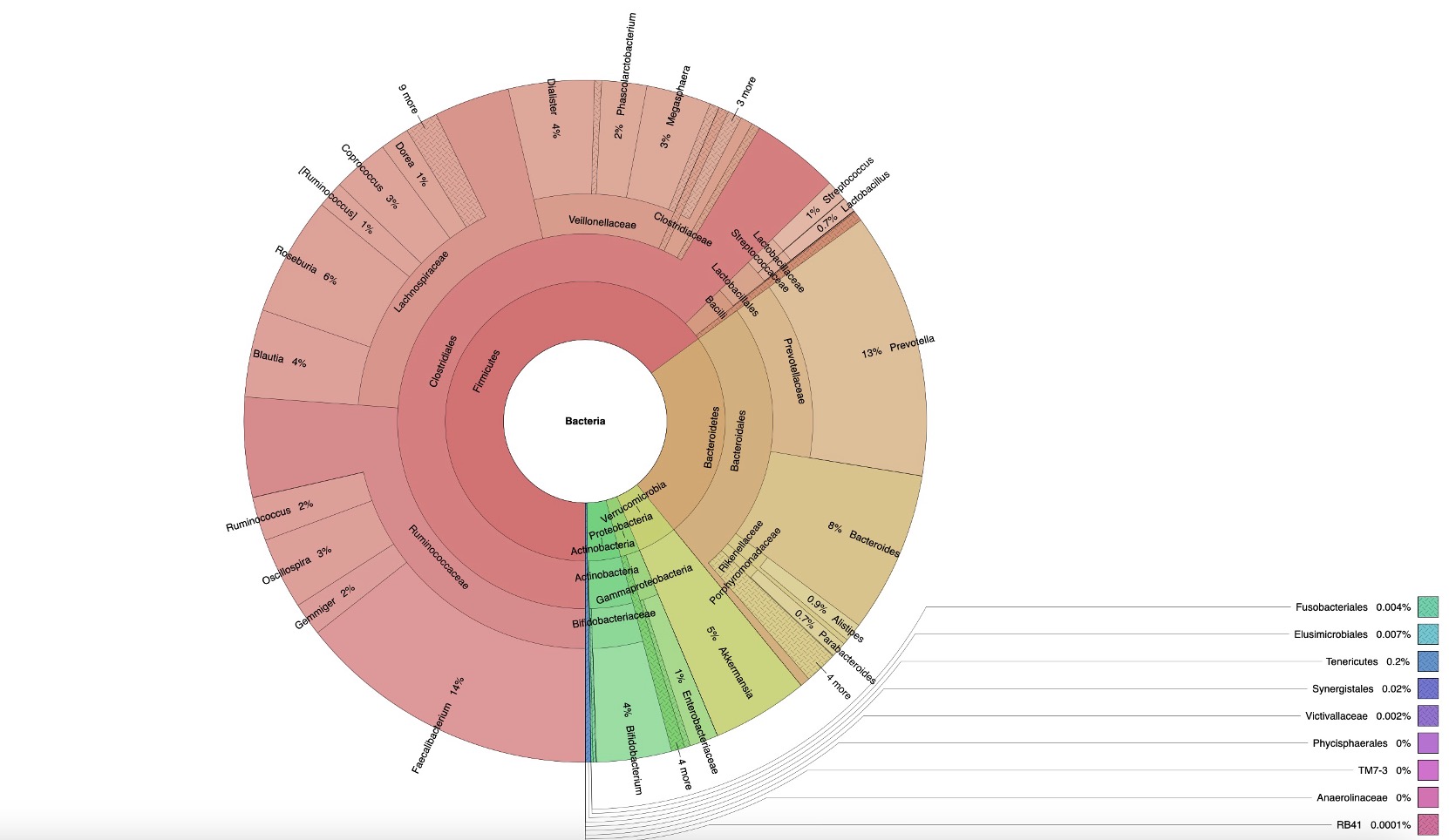

Supplement: Supplementary Figure 4 — Krona charts showing bacterial relative abundance in control group. [file Image_4.jpeg]
